# Accurate Diagnosis of Peritonsillar Abscesses Using Relative CT Number Measurements in Low-Density Areas of Contrast CT Images

**DOI:** 10.3390/jcm14124354

**Published:** 2025-06-18

**Authors:** Shu Kikuta, Takeshi Oshima

**Affiliations:** Department of Otolaryngology-Head and Neck Surgery, Faculty of Medicine, Nihon University, 30-1, Oyaguchi Kami-cho, Itabashi-ku, Tokyo 173-8610, Japan

**Keywords:** peritonsillar abscess, peritonsillar cellulitis, CT attenuation value, contrast-enhanced CT

## Abstract

**Objectives**: A diagnostic indicator for differentiating peritonsillar abscess (PTA) from peritonsillar cellulitis (PTC) has not been established. Our aim was to define radiological criteria for differentiating PTA from PTC. **Methods**: We retrospectively extracted low-density areas around the tonsils of PTA and PTC cases from contrast-enhanced CT images between 2021 and 2024. PTA cases were identified as those in which drainage by puncture or incision was observed, while PTC cases were those in which drainage was not observed. A total of 138 cases were finally analyzed (PTA, 111 cases; PTC, 27 cases). The CT attenuation value of a low-density area relative to that of the area surrounding the low-density area was used as the relative CT number, and relative CT numbers were compared between PTA and PTC cases. Using univariate and multivariate analyses, we identified factors that had diagnostic value for differentiating between PTA and PTC. **Results**: Relative CT numbers for PTA were significantly lower than those for PTC (*p* < 0.001). The univariate logistic regression analysis showed relative CT number, low-density ROI (region of interest), and surrounding ROI as having predictive value for differentiating PTA from PTC. In multivariate logistic regression analysis, only the relative CT number had predictive value for distinguishing PTA from PTC (odds ratio, 2.28), with a relative CT number of <0.46 being significantly associated with PTA. **Conclusions**: Low relative CT numbers could potentially be used to identify PTA, and their measurement could provide a diagnostic marker for the accurate diagnosis of abscess formation.

## 1. Introduction

A peritonsillar abscess (PTA) is defined as a segmental mass of pus that forms between the tonsillar capsule and the pharyngeal fascia, and it is the most frequent complication of acute tonsillitis [1]. If a PTA is not treated properly, it can spread to the adjacent deep cervical space, mediastinum, and cranial base [1,2], leading to sepsis, jugular vein thrombosis, and even airway obstruction and death [3]. Therefore, it is extremely important in clinical practice to accurately diagnose the presence or absence of abscess formation and to provide appropriate treatment without delay.

A PTA presents with general symptoms, such as sore throat, fever, and difficulty opening the mouth, but these symptoms also appear in peritonsillar cellulitis (PTC), which is probably the stage before PTA. Therefore, it is difficult to predict the presence or absence of an abscess based on these symptoms alone.

Imaging tests are very useful for differentiating between PTA and PTC and for accurately identifying the extent of the abscess [4]. In contrast-enhanced CT, the inside of the abscess is depicted as a low-density area, reflecting the presence of a large amount of fluid. In addition, one characteristic finding is the presence of a ring-shaped area of high density around the abscess, known as rim enhancement. However, interpretation of these findings can be subjective and can vary among observers, leading to limited diagnostic accuracy [5]. Moreover, a low-density area on CT does not always indicate an abscess, as other pathological processes can present with similar imaging features [6,7]. Given these limitations, relying solely on the subjective assessment of CT images may result in misdiagnosis and inappropriate management. Therefore, there is a clinical need for a more objective and quantitative method to evaluate low-density areas on contrast-enhanced CT scans. If such a method could accurately identify low-density areas that truly correspond to abscess formation, it would reduce diagnostic uncertainty, prevent unnecessary invasive procedures, and improve patient outcomes.

CT attenuation values (measured in Hounsfield units) are a measure of radiodensity [8]. The CT attenuation values assigned to each pixel represent the average linear attenuation coefficient of the corresponding voxel and primarily depend on the chemical composition of the tissue (e.g., −1000 HUs for air, 0 HUs for water, and 1000 HUs for bone). CT attenuation values can be affected by many variables, such as the scanner model, convolution kernel, reconstruction artifacts, beam hardening, and scanner linearity [9,10]. Therefore, they cannot be used as absolute values. We previously reported that the CT attenuation values of nasal tumors and fungal lesions could be differentiated from those of the brainstem and could be useful as a tool for distinguishing between individual tumor types [11,12].

In this study, the CT values of low-density areas relative to those in the areas surrounding the low-density areas were defined as relative CT numbers. We hypothesized that abscesses lacking vascular structures would be depicted as low-density areas on contrast-enhanced CT and that the relative CT numbers in PTA would be lower than those in PTC. We thus conducted a retrospective cohort study to investigate whether relative CT numbers could be used as a diagnostic marker for differentiating PTA cases from PTC cases.

## 2. Materials and Methods

### 2.1. Patient Selection

Patients who visited the Department of Otorhinolaryngology-Head and Neck Surgery at Nihon University Hospital between October 2021 and October 2024 and exhibited a low-density area around the tonsils on contrast-enhanced CT, performed because of a suspected PTA, were included in the study. These patients were identified through a review of the medical database.

A review of the medical database identified 182 patients with low-density areas around their tonsils on CT. Of the 182 patients, 44 were excluded for the following reasons: thirty-one because they had only non-contrast CT scans; three because their CT values could not be adequately measured due to dental artifacts; six because their low-density areas were too small to select ROIs from three slices; three because they did not undergo puncture or incision; and one because blood sampling had not been performed. After applying the inclusion and exclusion criteria, 138 cases were included in the final analysis (PTA, 111; PTC, 27). Among the 138 patients, 111 patients in whom puncture or incision resulted in the release of pus were analyzed as PTA cases, and 27 patients in whom puncture or incision did not result in the release of pus were analyzed as PTC cases. This study protocol was reviewed and approved by the Institutional Review Board of the Nihon University Itabashi Hospital (RK-250114-7).

Cases in which pus had been visibly drained by puncture or incision were defined as PTA cases, while cases in which no pus had been drained or in which the contents of the suction were only blood components were defined as PTC cases. To reduce the false negative rate due to puncture or incision, the procedure was performed by several doctors at least twice.

Demographic and clinical information was also recorded, including age, sex, duration of disease (the time from the onset of subjective symptoms to CT imaging, expressed in days), use of antibiotics prior to CT imaging, inflammatory status, body mass index (BMI), and Brinkman index (BI). Inflammatory status was assessed using white blood cell (WBC) and C-reactive protein (CRP) levels in blood samples collected on the same day as the contrast-enhanced CT. The BI is the number of cigarettes smoked per day multiplied by the number of years smoked.

### 2.2. Analysis of the CT Scan

CT scans were performed using a multi-detector CT scanner (Brilliance iCT, Philips Medical Systems, Best, The Netherlands). A non-ionic iodinated contrast agent (iopamidol, 300 mg iodine/mL; Iopamiron 300, Bayer Yakuhin, Ltd., Osaka, Japan) was administered intravenously at a dose of 1.5 mL/kg of body weight, with an injection rate of 1.5 mL/s. Image acquisition was initiated 90 s after the start of the injection. Images were reconstructed with a slice thickness of 1 mm using a soft tissue convolution kernel (Standard [B]).

The following characteristics of the lesions were analyzed using contrast-enhanced CT: low-density region of interest (low-density ROI, expressed in mm^2^), surrounding ROI (mm^2^), CT attenuation value (HU), and the presence or absence of a high-density zone (rim enhancement) around the low-density area. From the axial contrast-enhanced CT images, at least three slices were selected, and the ROI was set as large as possible within the range where the low-density area was completely contained within the boundary. The ROI was determined by an examiner who was blinded to the disease type and patient demographic information, and the CT attenuation values within the ROI were measured. The relative CT number was calculated by dividing the average CT attenuation value of a low-density area by the average CT attenuation value of an area surrounding the low-density area.

### 2.3. Statistical Analysis

Continuous and categorical variables are expressed as the mean ± standard deviation (SD) and number (%), respectively. Relative CT numbers were compared between PTAs and PTCs using the Mann–Whitney *U* test, because the data were not normally distributed. A *p*-value less than 0.05 was considered significant. Univariate and multivariate multiple regression analyses were performed to identify factors that predict relative CT numbers of low-density areas. Age, duration of disease, low-density ROI (mm^2^), surrounding ROI (mm^2^), WBC (×1000/µL), CRP (mg/dL), BMI, and BI were continuous variables. The predictors of sex (F, 0; M, 1), use of antibiotics before CT imaging (no, 0; yes, 1), disease type (PTA, 0; PTC, 1), and presence of rim enhancement (negative, 0; positive, 1) were considered to be dummy variables assigned a value of 0 or 1 to indicate the presence or absence of a categorical effect that could affect the results. The predictor variables were age, sex, duration of disease, use of antibiotics, disease type (PTA or PTC), low-density ROI, surrounding ROI, rim enhancement, WBC, CRP, BMI, and BI. The association between the predictor variables and the relative CT number was expressed as a univariate coefficient and the respective 95% confidence interval (95% CI).

A negative coefficient indicates an association with lower relative CT numbers, while a positive coefficient indicates an association with higher values. For dummy variables, the direction of the coefficient indicates the direction of association relative to the reference category. Specifically, for sex (F, 0; M, 1), a positive coefficient indicates that male sex is associated with higher relative CT numbers than those of female sex. For prior use of antibiotics (no, 0; yes, 1), a positive coefficient indicates that patients who had used antibiotics prior to CT imaging tended to have higher relative CT numbers than those who had not. For disease type (PTA, 0; PTC, 1), a positive coefficient indicates that PTC is associated with higher relative CT numbers than those of PTA. For rim enhancement (negative, 0; positive, 1), a positive coefficient suggests that the presence of rim enhancement is associated with higher relative CT numbers than those in its absence, whereas a negative coefficient suggests the opposite. Variables with a univariate *p*-value of <0.1 were included in the multivariate multiple regression analysis. A *p*-value of <0.05 was considered statistically significant.

Univariate and multivariate logistic regression analyses were also performed to identify factors that predict disease type (PTA or PTC). The predictive variables were age, sex, duration of disease, use of antibiotics before CT imaging, relative CT number, low-density ROI, surrounding ROI, rim enhancement, WBC, CRP, BMI, and BI. The association between the predictor variables and the disease type was expressed as the odds ratio (OR) and the respective 95% confidence interval (95% CI). Variables with a univariate *p*-value of <0.1 were included in the multivariate logistic regression analysis.

To evaluate intra-rater reproducibility of ROI placement, 20 randomly selected cases were re-examined by the same examiner two weeks later. The intraclass correlation coefficient (ICC) was calculated using a two-way mixed-effects model for absolute agreement. A 95% CI and a *p*-value were also reported to assess the statistical significance of the reliability. A *p*-value of <0.05 was considered statistically significant.

## 3. Results

Table 1 shows the demographic and clinical information (age, sex, duration of disease, blood sampling, rim enhancement, body mass index, and Brinkman index) of the 138 patients. No significant differences were detected between PTA and PTC cases in terms of demographic and clinical information.

**Figure 1:**
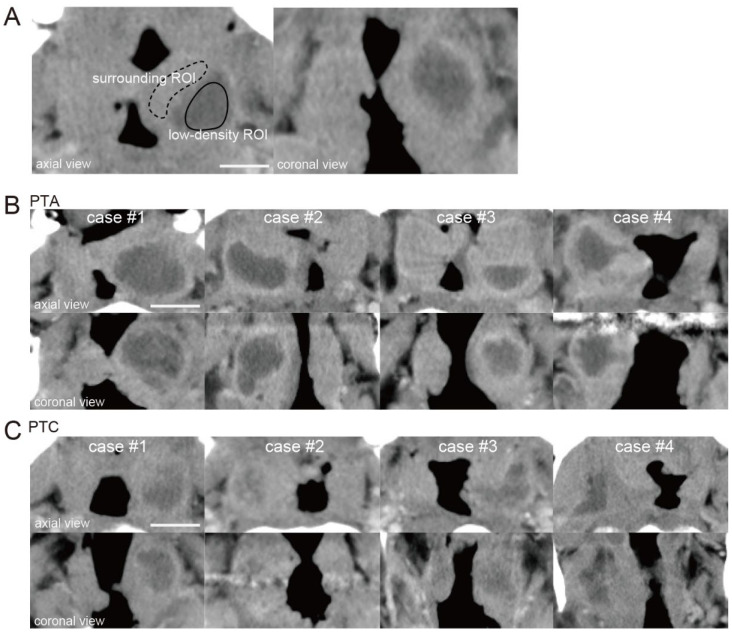
CT images of cases of peritonsillar abscess (PTA) and peritonsillar cellulitis (PTC). (**A**) ROI setting. The ROI was selected from the low-density area identified on the axial contrast-enhanced CT image (low-density ROI, solid circle). The surrounding area of the low-density area was set as the surrounding ROI (surrounding ROI, dotted circle). ROI, region of interest; scale bar, 20 mm. (**B**) Representative CT images of PTA cases (case #1 to #4). The upper images show axial sections, and the lower images show coronal sections. Scale bar, 20 mm. (**C**) Representative CT images of PTC cases (case #1 to #4). Scale bar, 20 mm.

The ROI for the low-density area (low-density ROI) was surrounded by a solid line, and the ROI for the surrounding area (surrounding ROI) was surrounded by a dotted line (Figure 1A). The relative CT number in the low-density area was calculated by dividing the CT number of a low-density area by that of an area surrounding the low-density area.

Figure 1B shows four representative PTA cases in which pus had been drained by puncture or incision. Figure 1C shows four representative PTC cases in which pus had not been drained. When the relative CT numbers of the low-density areas were measured in PTA and PTC cases, a slight difference was observed between the two (relative CT number; PTAs: case #1; 0.61, case #2; 0.64, case #3; 0.55, case #4; 0.57, PTCs: case #1; 0.39, case #2; 0.24, case #3; 0.35, case #4; 0.42).

**Figure 2:**
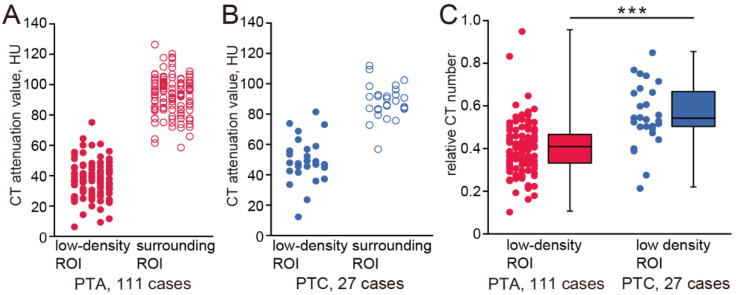
Relative CT numbers of PTAs and PTCs. (**A**) CT attenuation values of the low-density ROIs (red, closed circle) and surrounding ROIs (red, open circle) in PTA cases. Each point represents the CT attenuation values of the individual low-density ROI or surrounding ROI. (**B**) CT attenuation values of the low-density ROIs (blue, closed circle) and surrounding ROI (blue, open circle) in PTC cases. Each point represents the CT attenuation value of the individual low-density ROI or surrounding ROI. (**C**) Relative CT numbers in PTA cases and PTC cases. Each dot represents the relative CT number of an individual low-density area. All values are the mean ± SD. Relative CT numbers of PTAs were significantly lower than those of PTCs (Mann–Whitney test, *** *p* < 0.001).

The CT numbers of the low-density ROIs and surrounding ROIs of all cases are plotted in Figure 2A,B (PTA cases: low-density ROI, 37.24 ± 11.47, surrounding ROI, 93.18 ± 12.92; PTC cases: low-density ROI, 49.21 ± 14.94, surrounding ROI, 88.17 ± 11.31). The relative CT numbers in the low-density ROIs are shown in Figure 2C. The relative CT numbers of PTA cases were significantly lower than those of PTC cases (PTA, 0.39 ± 0.15; PTC, 0.55 ± 0.21, Mann–Whitney test, *p* < 0.001). The intra-rater reliability for ROI measurements was high, with an ICC of 0.92 (95% CI: 0.73–0.98, *p* < 0.001), indicating good consistency between repeated measurements.

In univariable analysis, disease type and low-density ROI were predictive of relative CT numbers (disease type: 95% CI, 0.99–2.07, *p* < 0.001; low-density ROI: 95% CI, −0.03–0.001, *p* = 0.03; Table 2). In multivariable logistic regression analysis, only disease type was predictive of relative CT numbers of low-density ROIs (95% CI, 0.93–2.01, *p* < 0.001, Table 2).

We searched for factors that had predictive value for distinguishing PTA from PTC by first conducting univariable logistic regression analysis to select appropriate parameters and then performing multivariable logistic regression analysis. In univariable analysis, relative CT number had predictive value in distinguishing between PTA cases and PTC cases (OR, 2.34, 95% CI, 1.59–3.44; *p* < 0.001, Table 3). In multivariable logistic regression analysis, including the factors relative CT number, low-density ROI, and surrounding ROI, PTA cases were significantly associated with low relative CT numbers (OR, 2.28; 95% CI, 1.53–3.38; *p* < 0.001, Table 3).

**Figure 3:**
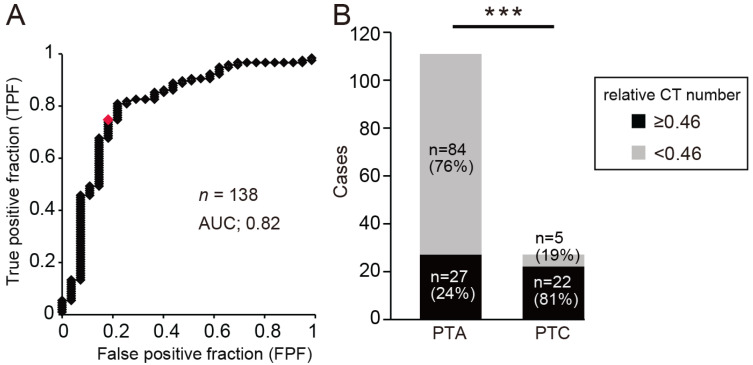
Receiver operating characteristic (ROC) curve analysis. (**A**) ROC curve after classification modeling was applied to the dataset. The curve was created by plotting the true positive rate (TPF, *y*-axis; sensitivity) against the false positive rate (FPF, *x*-axis; specificity) for various threshold settings. The red dot indicates the point closest to the top left corner on the curve, which represents the optimal threshold (sensitivity 76%; specificity 81%). (**B**) Comparison of relative CT values between PTAs and PTCs. Relative CT numbers of 0.46 or less were significantly associated with PTA detection. Chi-square test, *n* = 138, *** *p* < 0.01.

The optimum relative CT number cut-off value for determining PTA was calculated from the area under the receiver operating characteristic curve (AUC) as 0.46 (AUC, 0.82; OR, 2.28; sensitivity, 76%; specificity, 81%; Figure 3A). Most patients with a relative CT number of less than 0.46 had PTA, while most patients with a relative CT number of 0.46 or more had PTC (Figure 3B, chi-square test, *** *p* < 0.001). These results suggest that the difference in relative CT numbers may be related to the disease type and that PTA may be indicated when the relative CT number is low.

## 4. Discussion

In this study, we investigated whether radiological criteria based on contrast-enhanced CT could be used to differentiate between PTA and PTC. We defined relative CT number as the CT attenuation value of a low-density area relative to that of an area surrounding the low-density area and identified CT numbers that differentiated between PTA and PTC cases. The results showed that the relative CT numbers of PTA cases were significantly lower than those of PTC cases. Furthermore, multivariate logistic regression analysis showed that relative CT number alone had predictive value for differentiating PTA from PTC. These results indicate that differences in relative CT numbers are related to disease type and that low relative CT numbers may be diagnostic of PTA [13].

In peritonsillar infections, many bacterial infections are found in culture tests, and the main causative bacteria include *Streptococcus pyogenes*, *Fusobacterium necrophorum*, and *Streptococcus milleri* [1]. Therefore, treatment is mainly based on the administration of broad-spectrum antibiotics. If abscess formation is observed, then antibiotic treatment alone is insufficient, and additional procedures such as aspiration or incision are required [14]. However, these surgical procedures are associated with pain and bleeding, and they can cause a great deal of distress to the patient. In some cases, the abscess cavity is only a few millimeters away from the internal carotid artery, and thus, there is a risk of pseudoaneurysm formation due to damage to the arterial wall during aspiration or incision [15]. If it is possible to determine accurately in advance whether or not an abscess is present, then procedures that may cause pain or complications could be avoided.

In addition to clinical symptoms such as sore throat, difficulty swallowing, and fever, PTA cases include local symptoms such as swelling of the soft palate, deviation of the uvula to the unaffected side, and an exaggerated inflammatory response [4]. However, these findings are often observed in cases of PTC or acute tonsillitis, and the specificity of these symptoms is low at 50% [16]. Therefore, it may be difficult to accurately diagnose abscesses based solely on subjective symptoms, local findings, and blood sampling data and to differentiate between PTA and PTC.

Imaging tests are very useful for diagnosing abscesses [17,18]. Contrast-enhanced CT is frequently used for emergency imaging of the neck, but MRI and intraoral ultrasound have also been applied for this purpose [19,20].

MRI provides excellent contrast resolution for soft tissue, and it can differentiate reactive edematous changes from abscesses in the deep neck with high diagnostic accuracy [21,22]. The abscess cavity generally has a higher intensity than that of the tonsillar tissue itself on T2-weighted images, and it is also depicted with a higher apparent diffusion coefficient value. However, depending on the degree of inflammation and the cell density within the tonsillar tissue, these differences may not be apparent, and thus it is not easy to distinguish between tonsils and abscesses [21]. In addition, MRI takes longer to perform than CT and is not a suitable diagnostic tool for patients in the acute phase of a disease who need a quick diagnosis, especially if their condition is life-threatening. Furthermore, it cannot be used on patients with metal implants (such as pacemakers or artificial joints).

Ultrasound is a safe and non-invasive modality that avoids the health risks of radiation exposure. It is possible to make dynamic evaluations in real time, and changes in the size and shape of the abscess can be observed immediately [20]. However, when the abscess is located deep in the neck, it is difficult to assess its precise shape and extent [23]. In addition, if the abscess is located close to the bone, the hyperechoic bone surface results in strong reflection of sound waves that make the image unclear, and in cases where there is difficulty opening the mouth, the abscess cannot be observed from inside the mouth [16].

CT is a useful tool for evaluating abscesses and determining the extent of their development, as it can quickly produce high-resolution images. The center of the abscess appears as a low-density necrotic area, and the signal-to-noise ratio of the abscess and surrounding tissue can be increased by using contrast-enhanced CT [24]. However, if the low-density area is not clearly depicted, it is difficult to diagnose an abscess based on subjective observation alone. Objective evaluation of lesions on contrast-enhanced CT images may become a powerful diagnostic tool for detecting abscesses.

CT attenuation values are absolute values assigned to each voxel and represent the average linear attenuation coefficient of the corresponding voxel. CT attenuation values are mainly affected by the chemical composition of the tissue and individual organs [8]. There is controversy regarding the use of absolute CT attenuation values in the differential diagnosis of individual tumors because CT attenuation values vary depending on the settings used for image reconstruction. In previous studies, relative CT numbers, based on differences in CT values between a suspected disease area and a reference area, were reported to yield qualitative diagnoses of inflammatory diseases and tumors [12,25]. In this study, we used the relative CT number around an abscess and verified its usefulness for confirming the presence of an abscess. In a previous report, the false negative rate for detecting the presence of a suspected abscess was approximately 30% using CT images alone [5]. In this study, the false negative rate was approximately 20% (27/138 cases) based on the subjective evaluation of CT images alone. However, when the relative CT number was used and cases with an AUC value of 0.46 or less were selected, the false negative rate was approximately 5%, making it possible to diagnose PTA with relatively high reliability (sensitivity 76%; specificity 81%).

Changes in relative CT number may be related to differences in vascular structure and the degree of tissue edema. In PTA, the inflammatory response leads to increased vascular permeability and subsequent accumulation of fluid and necrotic debris, resulting in lower CT attenuation values [26]. Rim enhancement reflects the accumulation of inflammatory cells and neovascularization at the periphery of the abscess, where capillary permeability is increased due to cytokine release [27]. The central low-density area corresponds to necrotic tissue and pus, which have high water content and low vascularity [28]. These imaging characteristics have been described in prior studies of deep neck infections and cerebral abscesses [26,29]. In contrast, PTC typically shows less prominent changes in vascularity and tissue destruction, which may explain the relatively higher CT numbers and absence of rim enhancement [30].

In this study, we focused on PTA cases to determine whether relative CT number has diagnostic potential for the detection of abscess cavities. However, we believe that measuring relative CT number could also be used as an auxiliary diagnostic marker for diagnosing abscesses in other diseases and not only for deep head and neck infections.

This study has several limitations. First, the study had a retrospective cohort design and is thus subject to the inherent biases associated with retrospective studies. Second, because this study was conducted at a single medical institution, the generalizability of the study results is unknown. Third, although multiple aspirations or incisions were performed, none of them reached the abscess cavity, and as a result, there is a possibility that the number of PTA cases was underestimated. However, there was a significant difference in relative CT numbers between PTA and PTC cases. Therefore, we believe that relative CT number might be more useful than other diagnostic modalities for discriminating abscesses from other lesions.

## 5. Conclusions

PTA had significantly lower relative CT numbers than PTC. Only the relative CT number remained a significant predictor in differentiating PTA from PTC, with values < 0.46 strongly linked to PTA. These findings suggest that low relative CT numbers could be a useful marker for diagnosing PTA and abscess formation.

## Figures and Tables

**Table 1:** Patient demographic information for peritonsillar abscess cases and peritonsillar cellulitis cases (*n* = 138).

		PTA (111 Cases)	PTC (27 Cases)	*p*-Value
Age (years)		36.8 ± 15.9	37.0 ± 18.6	0.72
Sex (M/F)		69%/31%	70%/30%	0.92
Duration (days)		6.0 ± 5.1	6.5 ± 5.7	0.84
Blood sampling	WBC	14.5 ± 4.2	14.0 ± 4.1	0.79
	CRP	11.1 ± 7.6	9.7 ± 7.6	0.34
RE		Positive, 86 (77%)	Positive, 23 (85%)	0.38
BMI		23.3 ± 4.4	23.9 ± 2.5	0.33
BI		113.6 ± 49.6	74.5 ± 87.5	0.33

PTA, peritonsillar abscess; PTC, peritonsillar cellulitis; M, male; F, female; WBC, white blood cell; CRP, C-reactive protein; RE, rim enhancement; BMI, body mass index; BI, Brinkman index.

**Table 2:** Univariable and multivariable analysis of demographic and clinical factors predictive of relative CT numbers.

Variable		Univariable Coefficient (95% CI)	*p*-Value	Multivariable Coefficient (95% CI)	*p*-Value
Age (years)		−0.01 (−0.02–0.01)	0.76		
Sex (M/F)		−0.07 (−0.58–0.45)	0.8		
Duration (days)		0.004 (−0.04–0.05)	0.87		
Prior use or non-use of antibiotics		−0.27 (−0.8–0.27)	0.32		
Disease type (PTA or PTC)		1.53 (0.99–2.07)	<0.001	1.47 (0.93–2.01)	<0.001
Low-density ROI (mm^2^)		−0.01 (−0.03–0.001)	0.03	−0.01 (−0.02–0.003)	0.15
Surrounding ROI (mm^2^)		−0.01 (−0.03–0.01)	0.24		
RE		0.08 (−0.5–0.66)	0.79		
Blood sampling	WBC	0.004 (−0.05–0.06)	0.89		
	CRP	0.01 (−0.02–0.04)	0.53		
BMI		−0.01 (−0.06–0.05)	0.81		
BI		−0.01 (−0.002–0.0003)	0.2		

M, male; F, female; PTA, peritonsillar abscess; PTC, peritonsillar cellulitis; ROI, region of interest; RE, rim enhancement; WBC, white blood cell; CRP, C-reactive protein; BMI, body mass index; BI, Brinkman index; CI, confidence interval.

**Table 3:** Univariable and multivariable measures of factors predictive of disease type (PTA or PTC, *n* = 138).

Variable		Mean		Univariable OR (95% CI)	*p*-Value	Multivariable OR (95% CI)	*p*-Value
Age (years)		36.8 ± 16.4		1.01 (0.98–1.03)	0.95		
Sex (M/F)		M, 96 F, 42		1.05 (0.42–2.63)	0.92		
Duration (days)		6.1 ± 5.2		1.02 (0.94–1.1)	0.64		
Prior use or non-use of antibiotics		PTA	Use/no use, 62/49	0.61 (0.24–1.56)	0.31		
		PTC	Use/no use, 14/13				
Relative CT number		PTA	0.39 ± 0.15	2.34 (1.59–3.44)	<0.001	2.28 (1.53–3.38)	<0.001
		PTC	0.55 ± 0.21				
Low-density ROI (mm^2^)		PTA	37.24 ± 11.47	0.98 (0.94–1.0)	0.06	0.99 (0.965–1.03)	0.76
		PTC	49.21 ± 14.94				
Surrounding ROI (mm^2^)		PTA	93.18 ± 12.92	0.96 (0.93–1.01)	0.09	0.98 (0.92–1.04)	0.48
		PTC	88.17 ± 11.31				
RE		Positive, 109 (79%)		0.59 (0.19–1.89)	0.38		
Blood sampling	WBC	14.4 ± 4.2		0.97 (0.87–1.07)	0.54		
	CRP	10.8 ± 7.6		0.97 (0.92–1.03)	0.38		
BMI		23.5 ± 4.1		1.04 (0.93–1.15)	0.49		
BI		105.9 ± 238.6		0.99 (0.99–1.0)	0.45		

M, male; F, female; PTA, peritonsillar abscess; PTC, peritonsillar cellulitis; ROI, region of interest; RE, rim enhancement; WBC, white blood cell; CRP, C-reactive protein; BMI, body mass index; BI, Brinkman index; OR, odds ratio; CI, confidence interval.

## Data Availability

The datasets used during the current study are available from the corresponding author upon reasonable request.
